# Physiologic responses to exercise in survivors of critical illness: an exploratory pilot study

**DOI:** 10.1186/s40635-022-00461-8

**Published:** 2022-08-26

**Authors:** Matthew F. Mart, E. Wesley Ely, James J. Tolle, Mayur B. Patel, Nathan E. Brummel

**Affiliations:** 1 Division of Allergy, Pulmonary, and Critical Care Medicine, Department of Medicine, Vanderbilt University Medical Center, 1161 21st Avenue South, T1218 Medical Center North, Nashville, TN 37232 USA; 2 Critical Illness, Brain Dysfunction, and Survivorship (CIBS) Center, Vanderbilt University Medical Center, Nashville, TN USA; 3 Geriatric Research, Education, and Clinical Center (GRECC), Tennessee Valley Healthcare System, Nashville, TN USA; 4 Division of Acute Care Surgery, Department of Surgery, Section of Surgical Sciences, Vanderbilt University Medical Center, Nashville, TN USA; 5 Division of Pulmonary, Critical Care, and Sleep Medicine, Department of Medicine, The Ohio State University College of Medicine, Columbus, OH USA; 6 Davis Heart and Lung Research Institute, The Ohio State University Wexner Medical Center, Columbus, OH USA

**Keywords:** Critical illness, Cardiopulmonary exercise test, Post intensive care syndrome

## Abstract

**Background:**

ICU survivors suffer from impaired physical function and reduced exercise capacity, yet the underlying mechanisms are poorly understood. The goal of this exploratory pilot study was to investigate potential mechanisms of exercise limitation using cardiopulmonary exercise testing (CPET) and 6-min walk testing (6MWT).

**Methods:**

We enrolled adults aged 18 years or older who were treated for respiratory failure or shock in medical, surgical, or trauma ICUs at Vanderbilt University Medical Center (Nashville, TN, United States). We excluded patients with pre-existing cardiac dysfunction, a contraindication to CPET, or the need for supplemental oxygen at rest. We performed CPET and 6MWT 6 months after ICU discharge. We measured standard CPET parameters in addition to two measures of oxygen utilization during exercise (VO_2_-work rate slope and VO_2_ recovery half-time).

**Results:**

We recruited 14 participants. Low exercise capacity (i.e., VO_2Peak_ < 80% predicted) was present in 11 out of 14 (79%) with a median VO_2Peak_ of 12.6 ml/kg/min [9.6–15.1] and 6MWT distance of 294 m [240–433]. In addition to low VO_2Peak_, CPET findings in survivors included low oxygen uptake efficiency slope, low oxygen pulse, elevated chronotropic index, low VO_2_-work rate slope, and prolonged VO_2_ recovery half-time, indicating impaired oxygen utilization with a hyperdynamic heart rate and ventilatory response, a pattern seen in non-critically ill patients with mitochondrial myopathies. Worse VO_2_-work rate slope and VO_2_ recovery half-time were strongly correlated with worse VO_2Peak_ and 6MWT distance, suggesting that exercise capacity was potentially limited by impaired muscle oxygen utilization.

**Conclusions:**

These exploratory data suggest ICU survivors may suffer from impaired muscular oxygen metabolism due to mitochondrial dysfunction that impairs exercise capacity long-term. These findings should be further characterized in future studies that include direct assessments of muscle mitochondrial function in ICU survivors.

## Background

Over half of intensive care unit (ICU) survivors suffer from impaired physical function, including poor exercise capacity, for months to years after their critical illness, and this impairment is not fully explained by persistent muscle weakness [1–3]. Importantly, reduced exercise capacity is associated with greater mortality and loss of functional independence [4, 5]. To date, studies of physical rehabilitation interventions following an ICU stay have demonstrated mixed benefit for improving physical function [6, 7]. Thus, no effective means exist to rehabilitate or restore physical function for the millions who survive critical illness worldwide each year.

An important step in developing the next generation of interventions to improve outcomes is clarifying the mechanisms that underlie the persistent impairments in physical function after critical illness. Cardiopulmonary exercise testing (CPET) with gas exchange measurement is the reference standard for identifying cardiopulmonary or muscular etiologies of exercise limitations and impaired physical function [1, 8, 9]. To date, few studies have applied CPET to ICU survivors. Data from these studies demonstrate significant reductions in exercise capacity, which have been variably attributed to deconditioning and/or muscle weakness [1, 10]. Nevertheless, these studies have not used measures of whole-body oxygen metabolism and utilization available with CPET, suggesting our understanding of reduced exercise capacity in ICU survivors is incomplete. Moreover, most have enrolled populations with long durations of mechanical ventilation (i.e., 14 days or more) or a single disease process, such as coronavirus disease 2019 (COVID-19) [10–12]. Thus, a more robust characterization of exercise limitation in a general population of ICU survivors is needed. Identifying potential mechanisms common to critical illness that impair physical recovery would allow for further mechanistic research and potentially targeted therapies.

To address these knowledge gaps and investigate potential mechanisms of exercise limitation after critical illness, we conducted a pilot study of CPET and 6-min walk testing (6MWT) in ICU survivors without cardiac dysfunction. We aimed to characterize exercise limitations using both standard parameters and novel measures of oxygen metabolism.

## Methods

### Setting and participants

We enrolled adults, aged 18 years or older, who were treated for respiratory failure (defined as need for mechanical ventilation) and/or shock (defined as need for vasopressors) from medical, surgical, and trauma ICUs at Vanderbilt University Medical Center (Nashville, TN, United States) and enrolled in the parent “Measuring Outcomes of Activity in Intensive Care” (MOSAIC) prospective, observational cohort study (Clinicaltrials.gov identifier: NCT03115840). For this nested cohort study, we further restricted inclusion to participants without pre-existing or newly acquired cardiac dysfunction, contraindication to CPET [9], or need for resting supplemental oxygen at the time of follow-up. We also excluded patients with a primary admission diagnosis of COVID-19. The Vanderbilt Institutional Review Board approved the protocol (IRB Number 161157).

### Exercise testing

Participants completed an incremental CPET using a cycle ergometer (Lode Corival Recumbent Ergometer, Groningen, The Netherlands) with breath-by-breath gas exchange measurement (MGC Diagnostics Ultima CardioO2 metabolic cart, MGC Diagnostics, Saint Paul, MN, USA) and continuous non-invasive blood pressure, pulse oximetry, and electrocardiographic monitoring at 6 months [4.5–7 months] after ICU admission. On the same day, participants also completed 6MWT using standard protocols [13].

We calculated standard CPET measures including peak oxygen consumption (VO_2Peak_) using methods described by Wasserman and colleagues [9]. We a priori defined reduced exercise capacity as a VO_2Peak_ < 80% predicted for age and sex [9]. We also measured oxygen uptake efficiency slope (OUES), an effort-independent measure of exercise capacity that reflects the relationship between oxygen utilization and total ventilation [14]. Lower OUES, in the absence of reduced cardiac output, suggests inefficient oxygen utilization necessitating greater ventilatory effort to meet oxygen demand during exercise [14, 15]. We measured two additional parameters of oxygen utilization: VO_2_-work rate slope [16] and VO_2_ recovery half-time [17, 18]. The VO_2_-work rate slope measures the efficiency of aerobic energy generation with increasing physical effort during incremental CPET with lower values indicating worse oxygen utilization in exercising muscle [16]. VO_2_ recovery half-time is the time required for oxygen consumption to decrease to half of its peak value and is a marker of oxygen-dependent energy generation in muscle [18, 19]. Longer half-times indicate less efficient oxygen utilization during mitochondrial oxidate phosphorylation, which restores high energy phosphocreatine stores in muscle that were used at the start of exercise [17].

### Statistical analysis

Continuous data are presented using medians (interquartile range) and minimum to maximum ranges. Categorical data are presented as counts and percentages. Correlation analyses were conducted using Pearson’s product-moment correlation analysis, and Fisher’s Exact Test was used to compare categorical data in participants with reduced VO_2Peak_ versus normal VO_2Peak_. *p* values of < 0.05 were considered statistically significant. We used STATA (Version 16.1; StataCorp LLC, College Station, TX, United States) for all analyses.

## Results

**Fig. 1:**
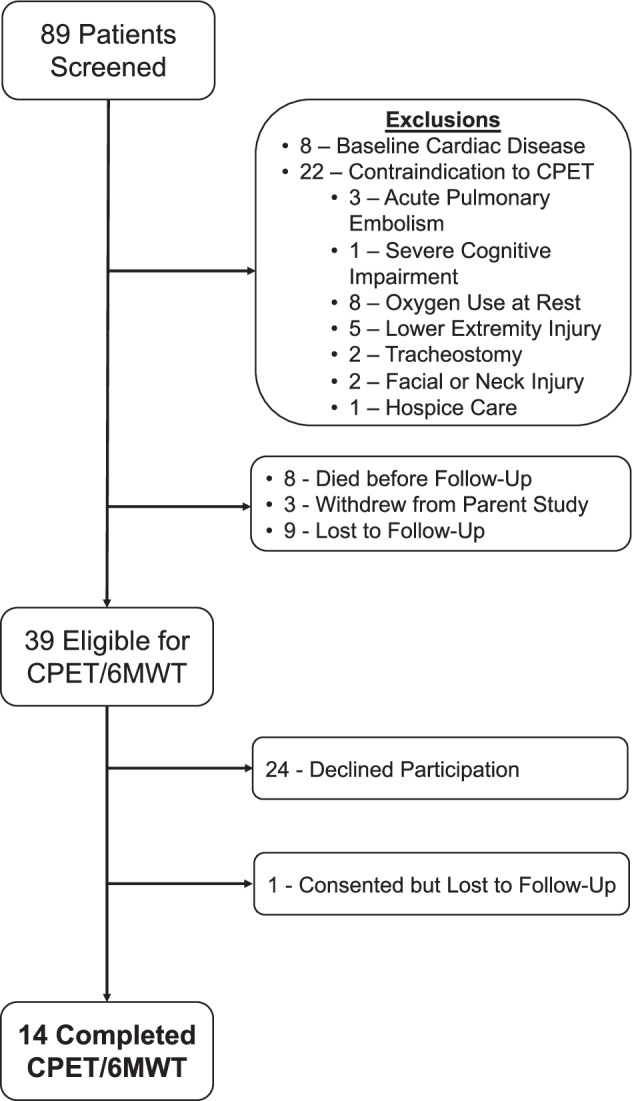
Consort diagram

Between October 2019 and June 2021, including a 6-month enrollment pause due to institutional COVID-19-related restrictions on the conduct of research using CPET, we screened 89 eligible ICU survivors (Fig. 1). Of these, 30 were excluded due to medical contraindications to CPET or pre-existing cardiac disease. An additional 8 participants died after discharge, 3 withdrew from the parent study, and 9 were lost to follow-up. Thus, 39 ICU survivors were eligible for this pilot nested cohort study. Of these, 24 patients declined participation and 1 participant was consented but was lost to follow-up prior to assessment. Thus, there were 14 participants who completed CPET and 6MWT.

At study enrollment, participants were a median age of 63, 53% were female, and had a median Sequential Organ Failure Assessment (SOFA) score of 10 (8–11) at ICU admission. The most common comorbidity present was diabetes mellitus (4 out of 14, 29%) followed by chronic liver disease (3 out of 14, 21%) and chronic respiratory disease (2 out of 14, 14%). All participants required mechanical ventilation for a median of 2 days [1–3], and 6 out of 14 (43%) were treated for shock at admission (Table 1). The median ICU length of stay was 5 days (3–8).

**Table 1:** Cohort characteristics (*N* = 14)

Variable	Median [IQR] or (%)
Age, years	63 [50–76]
Female, *n* (%)	8 (53%)
Body Mass Index (BMI)	29 [24–33]
Comorbidities	
Chronic respiratory disease^*^	2 (14%)
Chronic liver disease	3 (21%)
Chronic kidney disease	1 (7%)
Diabetes Mellitus	4 (29%)
Cerebrovascular disease or dementia	2 (14%)
History of malignancy	3 (21%)
Charlson Comorbidity Index	3 [0–6]
Duke Activity Status Index Score at ICU Admission^†^	25.8 [20.8–43.4]
Estimated VO_2Peak_ (ml/kg/min) at ICU admission^‡^	20.7 [18.5–28.2]
Mechanical ventilation, *n* (%)	14 (100%)
Duration of mechanical ventilation, days	2 [1–3]
Need for vasopressors at ICU admission	6 (43%)
SOFA Score at ICU Admission	10 [8–11]
ICU length of stay, days	5 [3–8]
ICU type, *n* (%)	
Medical	6 (43%)
Surgical	5 (36%)
Trauma	3 (21%)

^*^Includes chronic obstructive pulmonary disease and asthma

^†^Measured using the Duke Activity Status Index (Hlatky MA, et al. *Am J Cardiol* 1989;64:651–654)

^‡^Estimated using Duke Activity Status Index score conversion: Estimated VO_2Peak_ = 0.43 × DASI + 9.6

**Fig. 2:**
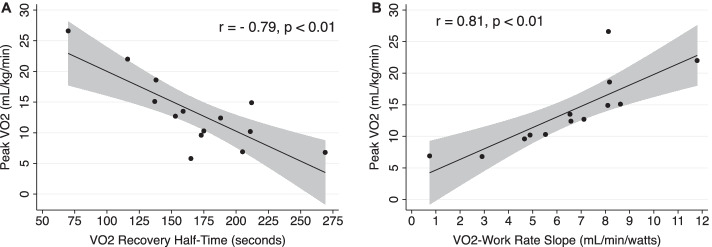
Correlation plots of oxygen utilization measures and peak oxygen consumption during cardiopulmonary exercise testing. Measures of oxygen utilization during exercise and in recovery in cardiopulmonary exercise testing (CPET) are strongly associated with peak oxygen consumption. **A** Demonstrates the correlation between peak oxygen consumption (VO_2Peak_) and VO_2_-work rate slope. VO_2_-work rate slope quantifies the change in oxygen consumption as work increases during cycle ergometry. Lower values indicate less efficient oxygen consumption and poorer aerobic metabolism (i.e., mitochondrial dysfunction). **B** The relationship between VO_2Peak_ and VO_2_ recovery half-time_._ VO_2_ recovery half-time quantifies oxygen consumption during recovery after exercise as skeletal muscle creatine is re-phosphorylated by mitochondria via aerobic metabolism. Longer durations of VO_2_ recovery half-time indicate impaired oxygen utilization and slower re-phosphorylation (i.e., mitochondrial dysfunction). All correlations calculated using Pearson’s correlation coefficient

**Fig. 3:**
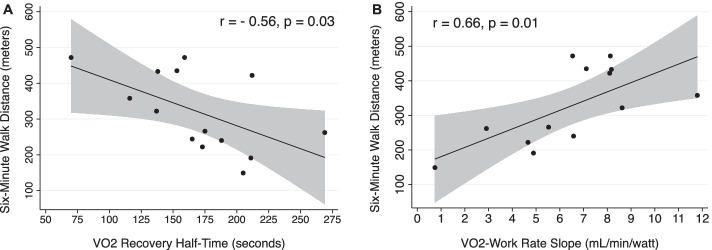
Correlation plots of oxygen utilization measures and 6-min walk test. Measures of oxygen utilization during exercise and in recovery in CPET are strongly associated with distance walked (m) during 6-min walk test. **A** and **B** Demonstrate correlations between 6-min walk distance and VO_2_-work rate slope and with VO_2_ recovery *t*_1/2_. All correlations calculated using Pearson’s correlation coefficient

At 6-month follow-up, the median VO_2Peak_ was 12.6 ml/kg/min [9.6–15.1 ml/kg/min] and the median 6MWT distance was 294 m [240–433 m] (Table 2). Using our a priori defined cutoff of VO_2Peak_ < 80% predicted [9], low exercise capacity was present in 11 out of 14 (79%). The median OUES was 65% predicted [54–73%], indicating that low exercise capacity was not related to participant effort. There were no differences between those with low VO_2Peak_ and those with normal VO_2Peak_ with regards to age (*p* = 0.68), sex (*p* = 0.20), shock at ICU admission (*p* = 0.52) or pre-ICU Duke Activity Status Index score (a measure of estimated exercise capacity, [*p* = 0.11]). No participants had ventilatory or gas exchange limitations to exercise nor exercise-induced ischemia. Participants did, however, have a pattern of low oxygen pulse, elevated chronotropic index, low VO_2_-work rate slope, and prolonged VO_2_ recovery half-time, indicating impaired oxygen utilization with a hyperdynamic heart rate response (Table 2), a physiologic pattern that mirrors responses seen in patients with mitochondrial myopathies [8, 15, 20]. VO_2_-work rate slope and VO_2_ recovery half-time were strongly correlated with both VO_2Peak_ and 6MWT distance (Figs. 2 and 3), suggesting that low exercise capacity may be related to skeletal muscle oxygen utilization.

**Table 2:** Physiological responses to exercise at 3-month follow-up (*N* = 14)

Variable	Median [IQR]
Peak or total exercise measures during cardiopulmonary exercise test (CPET) and 6 min walk test	
VO_2Peak_ (ml/kg/min)	12.6 [9.6–15.1]
% Pred. VO_2Peak_	51% [42–74%]
Respiratory exchange rate	1.1 [1.0–1.3]
OUES	1290 [1110–1428]
% Pred. OUES	65% [54–73%]
Peak work (W)	71 [53–97]
Peak heart rate (beats/min)	128 [109–143]
% Pred. heart rate	78% [66–83%]
O_2_ Pulse (ml VO_2_/heart rate)	8 [7–10]
% Pred. O_2_ pulse	72% [54–100%]
Chronotropic Index	1.35 [0.76–1.8]
VO_2_/work rate (ml/min/W)	6.6 [4.9–8.2]
VO_2_ recovery *t*_1/2_ (s)	169 [138–205]
Ventilatory reserve	43% [28–56%]
6-min walk distance (m)	294 [240–433]
Anaerobic threshold measures during CPET (*N* = 14)	
VO_2_-AT	10.9 [9.1–12.4]
% Pred. VO_2_-AT	38% [32–55%]
*V*_E_/VCO_2_ at AT	29 [25–35]

*VO*_*2*_ oxygen consumption, *VO*_*2peak*_ peak oxygen consumption, *% Pred.* percent predicted, *RER* respiratory exchange ratio, *HR* heart rate, *OUES* oxygen uptake efficiency slope, *AT* anaerobic threshold, *VO*_*2*_*-AT* peak oxygen consumption at anaerobic threshold

## Discussion

In this exploratory study of mechanisms underlying low exercise capacity in survivors of critical illness, we used cardiopulmonary exercise testing and novel measures of muscular oxygen metabolism and found the majority had significantly reduced exercise capacity with exercise responses that paralleled those seen in those with mitochondrial myopathies [8, 20]. We also found that measures of impaired muscular oxygen metabolism correlated strongly with both VO_2Peak_ and 6MWT, suggesting that exercise capacity in ICU survivors may be limited by oxygen utilization. These exploratory data indicate that impaired oxygen utilization due to mitochondrial dysfunction may contribute to persistent impairments in physical function following ICU hospitalization.

Our findings build upon prior studies of CPET in ICU survivors. Benington and colleagues recruited 50 survivors who had been treated with mechanical ventilation for 5 days or more and performed CPET at 6 weeks after hospital discharge [10]. They showed that 56% of these survivors had reductions in VO_2Peak_. Because the mean respiratory exchange rate (RER) at peak exercise was less than 1, they attributed these reductions in exercise capacity to participants not reaching anaerobic threshold due to deconditioning [10]. In contrast, we conducted exercise testing at 6 months after hospitalization in a cohort of ICU survivors with a median duration of mechanical ventilation of only 2 days and found that 11 out of 14 had reduced exercise capacity. Our participants, however, achieved a median RER > 1.05, indicating that the reduced VO_2Peak_ was not due to submaximal effort. In addition, we used an effort independent measure of exercise capacity (OUES) which was below age and sex predicted normal values in a majority of participants, suggesting that exercise limitations in our cohort were not attributable to only deconditioning. Our findings suggest that reduced exercise capacity following critical illness can be severe and persist beyond the initial recovery period, and careful evaluation of survivors with detailed CPET measurements of oxygen utilization may be valuable in characterizing exercise limitations in future studies.

Our findings also complement prior work by Van Aerde and colleagues, who recruited 313 survivors of critical illness and conducted CPET between 1 and 5 years after hospitalization. They found that 38% of survivors had impaired exercise capacity as measured by VO_2Peak_ [1]. Because cardiac and pulmonary limitations were not present, the authors pragmatically attributed exercise limitation to a muscular etiology. Our findings extend these through our use of specific measures of muscle oxygen utilization, VO_2_-work rate slope and VO_2_ recovery half-time. We found these measures to be strongly correlated with exercise capacity measured by both CPET and 6MWT, suggesting that impairments in muscle oxygen metabolism may play a role in reduced exercise capacity in ICU survivors.

Our finding that the global pattern of exercise responses, which included impaired oxygen utilization, increased ventilation, and hyperdynamic heart rate, parallels CPET findings seen in those with mitochondrial myopathies [8, 15, 20] also contributes to existing literature regarding physical function after the ICU. Muscle mitochondrial dysfunction occurs early in critical illness and sepsis [21], and it is strongly linked to survival. Few studies, however, have evaluated mitochondrial function in ICU survivors. Dos Santos and colleagues performed muscle biopsies in 11 patients at both 7 days after ICU discharge and 6 months after critical illness [22]. They found that mitochondrial content (i.e., number of mitochondria) was reduced at 7 days after ICU discharge but had normalized by 6*-*month follow-up. They did not, however, measure mitochondrial function (i.e., oxidative phosphorylation). We used novel CPET measures and found impaired muscle oxygen utilization, suggesting reduced mitochondrial function may be present. Because mitochondrial function may be impaired, even in the setting of a normal number of mitochondria [23], together our findings suggest that further study of mitochondrial function using both in vitro (e.g., muscle biopsies) and in vivo (e.g., 31-phosphorus magnetic resonance spectroscopy) techniques in survivors of critical illness is warranted.

Our preliminary findings that impaired exercise capacity may be related to impaired muscle oxygen utilization has potentially important relevance in improving physical recovery after critical illness. While prior post-ICU rehabilitation studies have predominantly utilized either self-directed exercise or protocolized exercise [7], our data suggest that future exercise rehabilitation studies could investigate novel rehabilitation approaches aimed at optimizing mitochondrial function [24] or be paired with mitochondrially targeted nutritional or pharmaceutical therapies [25, 26]. By further understanding the mechanisms by which exercise capacity remains impaired among ICU survivors, future interventions may be “metabolically tailored” to target the underlying pathophysiology that drives long-term impairments.

Our exploratory study has several strengths. We employed gold standard CPET to investigate potential mechanisms of exercise limitation and utilized novel measures of oxygen utilization not previously investigated in ICU survivors, finding that oxygen utilization is significantly associated with exercise capacity using two complementary assessments (VO_2Peak_ and 6MWT distance). Unlike prior studies of CPET who enrolled those with a mean duration of mechanical ventilation > 15 days [25], our participants were treated with mechanical ventilation for a median of 2 days, which is more reflective of a general ICU population and acute critical illness [27] rather than a persistent or chronic critical illness cohort [28]. The short duration of mechanical ventilation also suggests that reductions in exercise capacity may occur rapidly during critical illness, a finding in need of further study.

Our pilot study should also be interpreted considering several limitations. The sample size is small, and we did not include an external control group. Nevertheless, we compared CPET and 6MWT data to widely accepted predicted normative values adjusted for age and sex, making our results exploratory. We also cannot completely rule out impairments in cardiac or microcirculatory function without invasive CPET (e.g., Swan-Ganz and/or femoral catheterization during CPET). Nevertheless, we did exclude participants with known cardiac dysfunction and the routine use of invasive CPET does not reflect clinical practice. Finally, we did not measure muscle mitochondrial function directly using muscle biopsies or other techniques, such as 31-phoshporus magnetic resonance spectroscopy. Nevertheless, we applied validated CPET measures of muscular oxygen utilization that correlate with mitochondrial dysfunction in other clinical populations [20].

## Conclusions

In this exploratory study of survivors of critical illness, the majority of patients had impaired exercise capacity and low impaired muscular oxygen metabolism, suggesting impaired mitochondrial function. Future studies are needed to validate these findings and should measure mitochondrial function so that underlying mechanisms of impaired exercise capacity can be better understood.

## Data Availability

The data sets used and/or analyzed during the current study are available from the corresponding author on reasonable written request.
